# Guidelines for responsible short-term global health activities: developing common principles

**DOI:** 10.1186/s12992-018-0330-4

**Published:** 2018-02-07

**Authors:** Judith N. Lasker, Myron Aldrink, Ramaswami Balasubramaniam, Paul Caldron, Bruce Compton, Jessica Evert, Lawrence C. Loh, Shailendra Prasad, Shira Siegel

**Affiliations:** 1 Department of Sociology and Anthropology, Lehigh University, Bethlehem, PA 18015 USA; 2 Medical and Surgical Skills Institute, 2440 Westbrook Dr. NW, Grand Rapids, MI 45904 USA; 3 Swami Vivekananda Youth Movement, CA-2, KIADB Industrial Housing Area Ring Road, Hebbal, Mysuru, Karnataka State 570016 India; 4 University of Arizona College of Medicine, Phoenix, 4550 E. Bell Rd, Suite, Phoenix, AZ 172 USA; 5 Catholic Health Association of the United States, 4455 Woodson Road, St. Louis, MO USA; 6 University of California, 1001 Potrero Ave., 83, San Francisco, CA 94110 USA; 7 Dalla Lana School of Public Health, c/o Public Health and Preventive Medicine Residency Program, University of Toronto, 155 College Street, 5th Floor, MST 3M&, Toronto, ON Canada; 8 Center for Global Health and Social Responsibility, University of Minnesota, 420 Delaware Street SE, MMC 329, Minneapolis, MN 55455 USA; 9 Tel Aviv, Israel

**Keywords:** Short-term medical missions, Guidelines, Volunteers, Best practices, Global health education, Standards

## Abstract

**Background:**

Growing concerns about the value and effectiveness of short-term volunteer trips intending to improve health in underserved Global South communities has driven the development of guidelines by multiple organizations and individuals. These are intended to mitigate potential harms and maximize benefits associated with such efforts.

**Method:**

This paper analyzes 27 guidelines derived from a scoping review of the literature available in early 2017, describing their authorship, intended audiences, the aspects of short term medical missions (STMMs) they address, and their attention to guideline implementation. It further considers how these guidelines relate to the desires of host communities, as seen in studies of host country staff who work with volunteers.

**Results:**

Existing guidelines are almost entirely written by and addressed to educators and practitioners in the Global North. There is broad consensus on key principles for responsible, effective, and ethical programs--need for host partners, proper preparation and supervision of visitors, needs assessment and evaluation, sustainability, and adherence to pertinent legal and ethical standards. Host country staff studies suggest agreement with the main elements of this guideline consensus, but they add the importance of mutual learning and respect for hosts.

**Conclusions:**

Guidelines must be informed by research and policy directives from host countries that is now mostly absent. Also, a comprehensive strategy to support adherence to best practice guidelines is needed, given limited regulation and enforcement capacity in host country contexts and strong incentives for involved stakeholders to undertake or host STMMs that do not respect key principles.

**Electronic supplementary material:**

The online version of this article (10.1186/s12992-018-0330-4) contains supplementary material, which is available to authorized users.

## Background

In the last two decades, there has been a surge in international volunteering from many sectors, professions, and countries. Data from the US census and national surveys indicate that healthcare volunteering is a major portion of that growing volume [1–4]. Civilian and private sector organizations that sponsor these volunteer activities may be placed into four general categories: educational, faith-based non-governmental organizations (NGOs), secular NGOs, and for-profit companies.

Such overseas trips involving trained professionals, students, and lay volunteers have been described variously as volunteer missions, internships, global health education, and medical brigades. Short-term medical missions (STMM) and short-term experiences in global health (STEGH) represent the current most common shorthand. The former term tends to refer to service-oriented trips, while the latter is often used in discussion of educationally-focused experiences [5, 6], although these often include a service component. The term “mission” can lead one to conclude that these are religiously motivated, and many are, yet secular organizations often also refer to their activities as STMMs [3]. For the purpose of this paper, we include both service and educational types of short-term global health activities, sponsored by all types of organizations—NGO, corporate, university, and religious--and refer to them all for convenience as STMMs, recognizing that the term encompasses much more than religious missions and much more than physician-focused medical activities.

A growing body of literature enumerates real and potential harms of these largely unregulated activities. There are many problems that have been identified, often tied to the lack of control and direction by host organizations in defining the programs, the numbers of people volunteering, and the quality standards to be followed. Outside control and decision-making has led, often unwittingly, to displacement or devaluing of local providers, failure to arrange adequate follow-up of medical and surgical treatment, visitors carrying out medical procedures without adequate training and/or expertise, discordance between local needs and visitor offerings, inadequate licensing and credentialing of participants, inadequate intercultural aptitude of visitors, neocolonial attitudes towards host communities, inequitable use of funding, creation of dependency on external resources, and lack of continuity of services [7–9].

Existing guidelines are intended to offer guidance to organizations that are planning trips and/or to provide potential volunteers with questions to ask about organizations they are considering joining to participate in STMMs. The focus here is not on the technical requirements of specific clinical services but rather on general principles for ethical and responsible STMMs that would provide the most value and safest conduct for all concerned. A multiplying list of guidelines represent the desire of many to improve the quality of STMMs, but it is also essential to consider how desires and guidelines are translated into action.

It is important to note here that the use of the term “ethical” in many of the guidelines is not limited to the traditional bioethical model of four principles to be followed in patient care--respect for autonomy, nonmaleficence, beneficence, and justice [10]. Stone and Olson [11], for example, identify three areas of what they call “ethical concern” with regard to STMMs: context, limited time and resources, and cultural and language barriers. DeCamp [12] identifies seven “ethical principles” that should guide STMMs: establish a collaborative relationship, commit to benefits of social value, educate the local community and team members, build the capacity of local infrastructure, evaluate outcomes, and engage in frequent ethical review. While some guidelines focus on ethics in patient care, many guidelines in the global health context attempt to address a much broader set of issues in the relationship of volunteers and students to host communities.

This paper provides an overview of guidelines currently published, describing their intended audiences and the aspects of STMMs they address. It further considers how these guidelines relate to the desires of host communities, as seen in studies of host country staff who work with volunteers.

## Methods

We employed a “scoping review” methodology to identify published sources that provide guidelines for STMMs. The scoping review, which has increased in use in recent years, is “a form of knowledge synthesis that addresses an exploratory research question aimed at mapping key concepts, types of evidence, and gaps in research related to a defined area or field by systematically searching, selecting, and synthesizing existing knowledge” ([13], p. 1291). We followed the framework of authors who, drawing upon the essential work of Arksey and O’Malley and the Joanna Briggs Institute, have recommended five steps for such a review: identifying the research question, identifying relevant studies, study selection, charting the data, and collating, summarizing, and reporting the results [13, 14].

1. Identifying the research questions. As noted above, our purpose was to ask: who is creating guidelines for STMMs, what principles do they emphasize, how do they relate to the preferences of host communities, and how are they enforced?

2. Identifying relevant studies. We carried out an extensive review of academic and grey literature, relying on Medline, Google scholar, and Google searching of websites, using the keywords medical missions, global health, volunteering, guidelines, and standards. We supplemented this with the bibliographies of several articles that reviewed sets of published guidelines [4, 5, 14–16] and further drew on additional recommendations from colleagues, particularly for books and organizational websites.

3. Study selection. Our initial search cast a wide net and identified 144 articles or websites that either proposed standards for international short-term volunteer trips or critiqued volunteer practices and suggested improvements. We applied several inclusion and exclusion criteria to this list. We determined that for the purposes of answering the research questions, the guidelines had to 1) focus on trips that provide some type of health program rather than educational or other types of development programs; 2) be publicly available in a published journal article or website; and 3) include a specific set of principles to follow, beyond a mere critique of current practices in short term health trips. We excluded guidelines that were 1) limited to one specific aspect of a mission such as preparation or safety [17, 18]; 2) focused on specific medical practice guidelines, clinical techniques or supplies needed for specialty missions, such as pediatric plastic surgery projects or dermatology clinics [5]; 3) based on one experience [19]; 4) proposed and not yet published, or 5) that were summarized in a letter to the editor [20]. Applying these inclusion and exclusion criteria resulted in a list of 27 published guidelines (see Table 1).

4. Charting the data. The 27 articles, websites, and books were incorporated into a spreadsheet with columns for type of authorship (e.g. academic faculty, NGO, professional association), intended audience, and for the elements included in their guidelines. Based on a review of the 144 initial sources, we developed a list of 24 elements that emerged as desirable features of STMMs, and we coded for the appearance of these elements in each of the 27 sources. Two co-authors (JL and SS) separately coded all 27 references both for the 24 elements and for affiliation of the first author (health professional school faculty or student; practitioner in private or hospital practice; professional association; NGO; social science faculty; hospital association; host country practitioner) and intended audience (sponsor organizations, potential volunteers, host organizations) (See Table 1 for list of guidelines by first author affiliation). The initial coding resulted in the addition of new elements and the combining of others, yielding a revised list of 26 elements. In a second round of coding, inter-rater reliability was 78%. The two coders reviewed all disagreements and resolved them satisfactorily, with the result that one element was eliminated as unclear and superfluous, and two (accuracy in marketing, religious mission) were eliminated because they were each mentioned by only one source. The remaining 23 elements are listed alphabetically in Additional file 1, with one or two examples of each.

**Table 1:** Guidelines by Affiliation of First Author

A. Health Professional School Faculty	
Chapin E, Doocy S. [49]	
Crump JA, J Sugarman, and the Working Group on Ethics and Guidelines for Global Health Training (WEIGHT). [24]	
Dacso M, Chandra A, Friedman H. [50]	
DeCamp M. [12]	
Dowell J, Merrylees N. [51]	
Maki J, Qualls M, White B, et al. [34] (MEDICAL STUDENT)	
Mitchell K, Balumuka D, Kotecha V. [22]	
Olenick P, Edwards J. [52]	
Stone G, Olson K. [11]	
Suchdev P, Ahrens K, Click E, et al. [53]	
Umapathi [21]	
B. Health practitioner	
Dowell J, Merrylees N [51]	
Kingham TP, Price RR, Casey KM, et al. [54]	
Landau S. [55]	
O’Callaghan M. [56]	
Wilson, J.W., Merry, S., Franz, W.B. [8]	
C. Professional Association	
American Academy of Physician Assistants [57]	
American Dental Education Association [58]	
Association of American Medical Colleges [59]	
Forum on Education Abroad [60]	
Grimes, CE, Maraka J, Kingsnorth AN, et al. [23]	
International Federation of Medical Students’ Associations [61]	
World Medical Association [62]	
D. Social science faculty	
Lasker, JN udith [7]	
Melby MK, Loh LC, Evert J, et al. [6]	
E. NGO	
AmeriCares [63]	
F. Hospital Association	
Catholic Health Association [64]	

Finally, we assessed whether each source discussed procedures for enforcement of guidelines and possible challenges to enforcement.

## Results

The fifth stage of scoping reviews is collating, summarizing and reporting the results. We did so in line with the initial research questions, as follows:

1. Who is creating guidelines for STMMs?

The first authors of the 27 guidelines were identified as: medical/nursing school faculty/student (10); health practitioner (in private or hospital practice) (6); professional association (6); NGO (2); social science faculty (2); hospital association (1). (See Table 1).

All of the first authors are located in the Global North; only one source’s first author [21] was not from North America or Europe but from Singapore, which is also a high-income country with practitioners and learners that volunteer in lower-income settings countries. Several articles [20, 22–24] were co-authored by medical professionals from host countries as well as from the Global North, although in each case the lead author was based in the US. Grimes et al. (2013) notably report a study that was “conducted on behalf of and endorsed by” several surgical associations, including the West African College of Surgeons and the College of Surgeons of East, Central, and Southern Africa [22].

It is also important to note the dominance of medical practitioners and associations as authors, despite the fact that most programs and volunteers are sponsored by individual churches and small agencies, both nonprofit and for-profit [7]. These groups are barely visible in the creation of guidelines, except insofar as some individual practitioners who authored guidelines have led or participated in faith-based or secular NGO programs.

The intended audience for these guidelines included organizers (24), volunteers (25), and host communities/leaders (5), with many sources addressing more than one audience.

2. What principles do they emphasize? Table 2 lists the 23 elements with the frequency of mentions in the guidelines, organized into five more general principles. The most frequently included elements (by approximately half or more) are:

1. the necessity of preparing volunteers/students before they travel (Recommended preparation includes e.g. cultural humility and an understanding of the history, culture, health needs, and language of their host country.)
2. the necessity of having a partnership with an organization based in the host country that collaborates over time in planning and carrying out the program
3. adequate supervision and setting of limits for students
4. sustainability in the form of capacity building and/or training of local staff
5. volunteer safety
6. ethical principles for patient care
7. needs assessment.
8. evaluation of impact on host community.

**Table 2:** Guideline elements, frequency of mention, and Derived Core Principles

Guideline elements	Element frequency	Derived core principles
Volunteer motivations	8	Appropriate recruitment, preparation and supervision of volunteers.
Recruitment of volunteers	6	
Preparation in cultural competency/language/cultural humility	23	
Adequate supervision and limits for students. Participants	17	
Volunteer safety	15	
Matching volunteers’ skills with community/placement needs	10	
Post-participation debriefing/re-entry support	5	
Pre-trip volunteer technical job skills preparation	4	
Partnerships, collaborations	19	A host partner that defines the program, including the needs to be addressed and the role of the host community in directing and teaching the volunteers.
Needs assessment	14	
Clear statement of goals/agreement on purpose	12	
Avoid replacing local staff and workers	7	
Mutuality of respect and learning between hosts/guests	6	
Sustainability: capacity building, training of local staff	16	Sustainability and continuity of programming
Continuity of program/care	10	
Multi-week stay	5	
Ethical principles for patient care	15	Respect for governance, ethical and legal practices.
Attention to legal and governance issues	12	
Appropriate use of equipment and drugs	9	
Logistics/specifics of planning	10	
Financial transparency	7	
Evaluation of impact on host community	13	Regular evaluation of programs for impact and revisions made accordingly, based on data and analysis
Student learning/volunteer benefit	9	

Of these eight most frequently cited topics, three focus on the volunteers’ preparation, safety, and supervision; five focus on host communities’ needs, resources, patient care, participation in the program, and the impact of programs.

3. How do they relate to the preferences of host communities? The inherent risk in identifying core principles lies in the finding that almost all guidelines have been published by individuals and organizations from the Global North, either as sponsors or as analysts of short-term health trips. Their audience is also primarily to be found among those who organize or volunteer for such trips.

Fortunately, there are a number of studies of host country staff and community members regarding their perceptions of STMMs which can be used to validate existing guidelines [7, 25–33]. The findings largely agree on several characteristics of the most and least desirable programs. Despite general satisfaction with and appreciation of outside groups, host community concerns focus primarily on volunteers’ lack of: cultural awareness and humility, leading to offensive behavior and attitudes of superiority; lack of real partnership, leading to suboptimal involvement of host partners in decision-making; absence of mutuality of learning in that volunteers de-value or do not focus on learning *from* hosts; absence of continuity of care; poor communication between hosts and volunteers; and lack of congruency between volunteers’ skills and communities’ needs and priorities. Additionally, they express concerns about the potential for competition with and even displacement of locally-trained professionals.

In comparing these results with the elements most commonly found in the guidelines, we see that partnership and volunteer preparation are prominent in both. But host community staff’s desire for mutual learning and avoidance of competition (both of which require respect for and acknowledgement of local expertise) are less frequently mentioned in the guidelines included in the review.

4. How are they enforced?

None of the selected guidelines for review mapped a plan for assuring adherence to the proposed elements. Several addressed the challenges of implementing guidelines or suggested avenues to do so. Maki et al. point out the lack of any international body to provide oversight of STMMs [34]. Stone and Olsen agree that “no central monitoring body exists…” [11]. Melby et al. (2016) call on professional associations to monitor short-term experiences for cultural and ethical concerns and make information on their quality available to members [6]. They suggest this “could include the use of assessment data to accredit STEGHs, develop uniform program standards (e.g., with respect to preparing trainees), and facilitate a paradigm shift that focuses on promoting participatory research and programming that prioritize elevating the voice and input of LMIC-based stakeholders… [There is a] need for objective data on effective STEGH models that positively influence community health outcomes… Effective deployment of online databases could allow the global health community to evaluate the ethics and sustainability of STEGHs.”

## Discussion

The field of STMMs is evolving, with increasing concerns about their utility and the potential for unintended consequences. The value of this study lies in the opportunity to distill and coalesce guideline elements in order to synthesize a set of core principles relevant to all short-term global health outreach efforts.

### Core principles

Based on the analysis of 27 guidelines, we can combine the elements identified into five central themes, each of which includes at least one of the eight most frequently mentioned elements. For a STMM to be maximally valuable, according to these guidelines, it should have at least these features (see Table 2):

1. Appropriate recruitment, preparation and supervision of volunteers
2. A host partner that defines the program, including the needs to be addressed and the role of the host community in directing and teaching the volunteers
3. Sustainability and continuity of programs
4. Respect for governance and legal and ethical standards
5. Regular evaluation of program impact on host community

### Host perspective

The addition of host perspective, based on limited studies to date (carried out almost entirely by outsiders), leads us to add to this list the necessity of mutuality and respect. As Melby et al. (2016) conclude in their analysis, which largely considers student experiences, “…the discourse around program implementation should refocus on STEGHs’ impact on host communities...STEGHs must address, rather than perpetuate, underlying power imbalances, ethical pitfalls, resource differentials, and inequities that the global health movement seeks to eliminate” [6].

These power imbalances are seen in the language often used to describe hosts-- “recipients” and “beneficiaries” --which assumes they are receiving something valuable and benefiting from a program, often without evidence. There is also an assumption among practitioners in global health that knowledge is created in the Global North to be received by those in the Global South. (Thus, students from HIC who visit LMIC are assumed by many to be “helping”, while even within direct exchange programs, students from LMIC visiting HIC are assumed to be “learning”.) Lack of attention to the assets and skills and experiences of host staff and communities reinforces these assumptions and disadvantages both visitors and hosts. Yet there is evidence that transfer of knowledge and experience in the opposite direction can benefit wealthier countries [35].

While hosts may feel bound by norms of hospitality, outside volunteers often ignore that there are norms for guests that they should observe, starting with respect, consideration, and gratitude [36]. In order for hosts to appreciate their visitors fully and for both parties to optimize the utility of short term encounters, respect and mutuality should be added as a sixth principle to the five core principles identified above.

### Core principle implementation

Although there are many efforts to improve STMMs by encouraging adherence to guidelines or principles, these efforts are not matched by mechanisms to incentivize adoption or adherence. Caldron et al., in their systematic literature review and in their study of physician volunteers, found no evidence for influence of sanctioning bodies or professional societies on the proliferation of STMMs nor on physician motivation [5, 37]. Little information is available to guide prospective volunteers or host partners toward quality programs through reputable organizations. Research and anecdotal reports challenge the claims of many sponsoring organizations that they have active host partners, that they properly prepare volunteers to understand the host country culture, or that they adequately monitor their activity [7, 38].

The absence of legal or professional oversight means that there are few financial or legal consequences for conducting irresponsible, even harmful programs. An exception is found in Ireland, where overseas development programs are vetted by Comhlamh, an NGO funded by the Irish government. Organizations that do not meet Comhlamh standards cannot receive government subsidies (website; personal communication). Similar efforts are underway in the EU (website; personal communication). These models are not present elsewhere, to our knowledge.

Host countries vary with regard to the legal requirements for visiting medical groups and enforcement of requirements. Ministries of Health often have strict regulations governing how physicians can qualify to practice in their countries. Ghana, for example, states that “It is against the law to practice in Ghana without being registered with the Medical and Dental Council Ghana, it is also unlawful to employ and engage the services of a practitioner who is not registered with the council” [39, 40]. Some countries, such as China, Philippines, and Belize, have regulations specific to the registration of foreign medical missions and the U.N. also has guidelines for foreign medical teams responding to disasters. These include requirements of registration, submission of credentials, and oversight by host organizations and legal authorities [41–44]. However, having rules does not mean that there exist the resources for their enforcement.

We were not able to locate any research on enforcement of such regulations. Research is needed on models for and experience with the application of these regulations; anecdotal reports suggest that many program providers and volunteers ignore them. Additionally, it may be difficult to navigate acceptable local compliance without the guidance of a host partner. Yet only 19 of the 27 guidelines call for a host partner, and research on actual practices indicates that as many as half of programs do not always work with host partners [7].

Several barriers to the adoption of and adherence to core principles exist in both sponsoring and host countries. Widespread beliefs on both ends about the supposed inherent goodness of volunteering and the assumed value to host communities make it very difficult to limit these activities and may be a barrier to gathering data on impact and effectiveness. The imposition of strict legislative regulations is likely to be resisted by those who fear it could deter participation from medical professionals as well as students [37].

Health volunteers typically have the legal standing of tourists with the basic responsibility to abide by the tort laws of host countries but without additional accountability for impacts or actual utility. Limited resources and more urgent priorities for governments of host countries may impede interest and capacity to monitor thousands of volunteer programs. There are also conflicting incentives for host country stakeholders; while short-term volunteer trips may undercut local services or disincentivize long-term investment by local authorities in rural healthcare, they also contribute to the local economy, temporarily filling large gaps in government services, and may improveing morale in communities with scarce resources. Finally, powerful cultural norms that give primacy to hospitality as well as hosts’ relative lack of power [45] may impede host communities’ expression of justified criticism of activities that cause problems or fail to meet the community’s needs and/or priorities.

Often, a lack of financial or legal incentives or restrictions leave volunteer organizations free to proceed per their own sense of what will work (or what they think has worked in the past) and in response to their own organizational imperatives, e.g. financial, reputational, or recruitment [46]. Several billion dollars per year are spent on STMMs, generating profits for companies, suppliers and airlines in the Global North and benefits for stakeholders on both sides of the exchange [3, 47]. Financial gains, often in the form of fees paid by volunteers, incentivize efforts to increase the number of volunteers and to minimize the expense of working with partners for planning and evaluation. Interestingly, the need for financial transparency was mentioned in only one-fourth of guidelines.

### Limitations of study

It is possible that this review may have missed some potentially valuable guidelines. Most importantly, less accessible guidelines and perspectives from host communities are more likely to be overlooked based on our methodology. However, these limitations are somewhat mitigated by the large concordance identified among themes. Also, the stated belief, among the guidelines reviewed, that STMMs must respect host communities suggests at least a verbal endorsement by guideline authors of the findings of host studies.

## Conclusion

The review of a body of aggregated guidelines and of studies of host community perspectives reveals a broad consensus on six core principles for effective and ethical STMMs. These are:

1. appropriate recruitment, preparation and supervision of volunteers
2. a host partner that defines the program, including the needs to be addressed and the role of the host community in directing and teaching the volunteers
3. sustainability and continuity of programs
4. respect for governance and legal and ethical standards
5. regular evaluation of programs for impact
6. mutuality of learning and respect for local health professionals

There remain significant barriers to implementing and enforcing guidelines, and evidence suggests that most are not observed in practice [48]. Multi-pronged strategies addressing these obstacles are needed to maximize the very real benefits of some STMMs and address the concerns and harms associated with those that are poorly conducted. A first step at eroding several of those barriers is defining a core set of fundamental principles distilled from the body of published guidelines, culled from multiple sources and applicable to all responsible short-term, cross-border health-related initiatives.

This study of published guidelines provides responses to the questions posed at the onset. Guidelines have been written primarily by academics, practitioners, and professional associations in the global north. There are indeed common elements that transcend the type of organization or volunteer that may be coalesced into core principles that are universally applicable to STMMs. Such standards encompass much of what has been learned from studies of the viewpoints of host communities in the Global South and not simply from the perspectives of the professionals from the Global North, but the former studies are more likely to emphasize the necessity of respect and mutuality. There is an almost total absence of evidence of the influence of guidelines on actual practices. Identifying overarching principles that can be turned into guidelines for STMMs is only a first step; substantial improvement of short-term global health activities requires regulations and incentives in both host and sponsoring countries.

## Additional file

Additional file 1: Appendix. Guideline Elements and Examples. (DOCX 19 kb)
