# Supplementary material for: Guidelines for responsible short-term global health activities: developing common principles
Source: Global Health. 2018 Feb 7;14:18. doi: 10.1186/s12992-018-0330-4 (PMC5803894; doi:10.1186/s12992-018-0330-4)
Supplement: Additional file 1: Appendix. — Guideline Elements and Examples. (DOCX 19 kb) [file 12992_2018_330_MOESM1_ESM.docx]

Appendix A. Guideline Elements and Examples

| Focus Area | Example Quotes | Source |
| --- | --- | --- |
| Adequate supervision and setting of limits for students | PAs should provide only those services for which they are qualified via their education and/or experiences. | American Academy of Physician Assistants |
| Appropriate use of equipment and drugs | Use medications available locally or through the WHO’s list of Essential Medicines | Wilson et al., p.615 |
| Attention to legal and governance issues | Meet licensing standards, visa policies, research ethics review, training on privacy and security of patient information, and other host and sending country requirements. | Crump, Sugarman, and WEIGHT, p.1180 |
| Avoid replacing local staff and workers | Care must be used to prevent undermining the trust that the local community has in its own surgical workforce…. A visitor’s role should be supportive, rather than substitutive or authoritative. | Kingman et al., p. 20 |
| Clear statement of goals/agreement on purpose | Establish clear goals and priorities by developing the type, frequency, timing and duration of programme. | Umapathi et al., p.4 |
| Continuity of program/care | There should be regular trips to the same institution over a number of years to ensure capacity building and sustainability.    Lack of secure access to an ongoing supply (of medications for chronic conditions) and inadequate follow-up for the patient is a recipe for iatrogenic mishaps. | Grimes et al., p. 1204          Olenik & Edwards, p. 206 |
| Ethical patient care | Practice good medicine. Remember: first, do no harm.    The quality of healthcare delivered within an underserved location should be the highest possible allowed by the resource constraints. The needs of the patients should always come first. | Landau, p.144      Wilson, Merry, & Franz, p.614 |
| Evaluation of impact on host community | Develop outcome assessments of patient care activities, patient safety, quality control, and overall mission impact. | Wilson et al., p.615 |
| Financial transparency | Recognize the true cost to all institutions (e.g., costs of orientation, insurance, translation, supervision and mentoring, transportation, lodging, health care, administration) and ensure that they are appropriately reimbursed. | Crump, Sugarman, and WEIGHT, p.1180 |
| Logistics/specifics of planning | Practical matters such as transportation, housing, meals, and security can directly impact effectiveness…the challenges of practicing in low resource settings and makeshift clinics can complicate even simple details, such as hygiene and provision for appropriate patient privacy…” | Olenik & Edwards, p. 208 |
| Matching volunteers' skills with community/placement needs | it is important to bring team members with diverse specialties … so that volunteers can use their specific strengths to address the community’s prevailing needs. | Suchdev et al. |
| Multi-week stay | Spending several weeks on a mission allows the visitor to develop rapport with local providers and trainees, contribute to (but not replace) local curricula, and perform operations with an appropriate interval of post-operative follow-up care. | Mitchell et al., p. 2 |
| Mutuality of respect and learning between hosts and guests | Host-community members want more than helpful visitors with skills and resources, … They want to be involved in the work programs undertaken by volunteer organizations, and they want to be respected. They want a relationship of equality in which each partner learns from and benefits from the other. | Lasker, p. 166 |
| Needs assessment | The trip must be responsive to local needs. There must be adequate contact prior to the trip between the visiting team and the local medical staff…. | Grimes et al., p.1204 |
| Partnerships/collabora-  tions | Partnership with an NGO, government agency, or other local organization determines the type and extent of work that can be done. Organizations that understand and work within the infrastructure of a community can facilitate the integration of medical and public health projects and assure their continuity. | Suchdev et al. |
| Post-participation debriefing for volunteers/Re-entry support | Ensure … reentry assessment and  feedback are available for all students. | Forum for Education Abroad, p. 2 |
| Preparation in cultural competence/language/cultural humility | Provide compulsory pre-departure training for students going on placements abroad that cover issues of … cultural awareness, language competencies, and ethical considerations    Adequate … respect and understanding of cultural differences are essential for patient safety and the success of the medical service trip. | IFMSA Policy Statement, p.4              Chapin and Doocy, p. 52 |
| Pre-trip volunteer technical skills preparation | Predeparture training and other extracurricular professional development is necessary preparation | Melby et al., p. 3 |
| Recruitment of volunteers | Is the application process adequate for credentialing, along with screening of competency and health of volunteers? | Olenik and Edwards, p. 206 |
| Student learning/ volunteer benefit | Personal growth does (and indeed should) accompany service in such environments…During these experiences, the focus and priority should be given to the patients, but the health care providers and the communities can and should both benefit. | Stone and Olson, p.241 |
| Sustainability: Capacity Building, training of local staff. | …long term solutions for these communities need to involve local infrastructure and human resource development to avoid dependence on a repetitive and often disjointed cycle of STEGHs.    A significant proportion of any trip should be on training local health providers. | Melby et al. p. 4      Grimes et al., p.1204 |
| Volunteer motivations | Ensure your motives are appropriate. | CHA, p. 6 |
| Volunteer safety | The sending institution should ensure that a travel safety policy is in place and that there is appropriate administrative accountability. | Dacso, Chandra, Friedman, pp. 1649-1650 |
